# The Possible Effects of High Vessel Traffic on the Physiological Parameters of the Critically Endangered Yangtze Finless Porpoise (*Neophocaena asiaeorientalis ssp*. *asiaeorientalis)*

**DOI:** 10.3389/fphys.2018.01665

**Published:** 2018-11-28

**Authors:** Ghulam Nabi, Yujiang Hao, Richard William McLaughlin, Ding Wang

**Affiliations:** ^1^Institute of Hydrobiology, Chinese Academy of Sciences, Wuhan, China; ^2^University of Chinese Academy of Sciences, Beijing, China; ^3^General Studies, Gateway Technical College, Kenosha, WI, United States

**Keywords:** acoustic pollution, cortisol, critically endangered, stress, thyroid, Yangtze finless porpoise

## Abstract

**Background:** Poyang is the largest freshwater lake in China, where the acoustic environment and space for the critically endangered Yangtze finless porpoises (YFPs) has been altered by heavy vessel traffic and dredging activities. The density of vessel and the rate of dredging increases annually, especially in the area with the highest density of YFPs. The heavy vessel traffic can cause an increase in the physical activities and direct physical injuries to the YFPs. Furthermore, noise is a potent stressor to all cetaceans irrespective of age and can compromise all their physiological functions. The objective of this study was to examine the possible effects of heavy vessel traffic and dredging on the biochemistry, hematology, adrenal, thyroid, and reproductive hormones of two different YFP populations. One population was living in Poyang Lake and the second living in the Tian-E-Zhou Oxbow which is a semi-natural resserve.

**Results:** The results showed statistically significantly higher levels of serum cortisol, fT3, fT4, and lowered testosterone in both adult and juvenile YFPs living in Poyang Lake vs. adult YFPs living in the Tian-E-Zhou Oxbow. The serum biochemical parameters (Aspartate Amino Transferase, Alkaline Phosphatase, High Density Lipoprotein cholesterol ratio, Globulin, Uric acid, Glucose, K^+^, and Amylase) and the hematology parameters (Red Blood Cells, Hematocrit, Mean Corpuscular Volume, White Blood Cells, and Eosinophils) were statistically significantly higher in the adult Poyang Lake YFPs vs. adult Tian-E-Zhou Oxbow YFPs. On the other hand, adult males of the Tian-E-Zhou Oxbow also showed significantly higher levels of the serum biochemical parameters (Total Cholesterol, Light Density Lipoprotein cholesterol, Direct Bilirubin, Albumin, Lactate Dehydrogenase, CO_2_, and Na^+^) and the blood parameters (Mean Corpuscular Hemoglobin and Mean Corpuscular Hemoglobin Concentration). In Poyang Lake YFPs, various parameters showed significantly positive (fT4, amylase, neutrophil, Ca^+2^) or negative (total protein, lymphocyte) correlations with cortisol levels.

**Conclusions:** The hyperactivity of adrenal glands in response to heavy vessel traffic and dredging resulted in significantly elevated cortisol levels in Poyang Lake YFPs. The higher cortisol level could possibly have affected various hormonal, hematological, and biochemical parameters, and ultimately the YFPs physiology.

## Background

For the economy of China, the Yangtze River is very crucial and is, therefore, a heavily trafficked waterway and inland water transport (Liu et al., 2000; Schelle, 2010). The vessels include motorized ferries, small boats, oil tankers, and container ships (Smith and Reeves, 2000). In the year 2000, ~400 million tons of cargo were transported on this river, which has increased to ~1.2 billion tons of cargo (Yang et al., 2009). In the 1980's, the vessel trafficking was low in the Yangtze River (Turvey et al., 2007). Since this river is the backbone and golden channel of the Chinese economy (Fu et al., 2010), the number of vessels increased up to 5 times in 2006 (Wang et al., 2006), and today, it is the world's busiest inland river (Lixin, 2018). To accommodate the increased ship traffic and to allow large container ships, the channel was widened using explosives (Wang, 2009).

The practice of intensive sand dredging has been banned in the Yangtze River since 1998 (Zhong and Chen, 2005). It restarted in 2001 in Poyang Lake, which is an appended lake of the Yangtze River (Zhong and Chen, 2005). The number of dredging vessels rapidly increased due to the potential profit (Wu et al., 2007). The number of vessels in Poyang Lake increased from zero in 2000 to ~400 in 2005 (Wu et al., 2007). Even today there are a few thousand vessels engaged in digging and transportation (Wang et al., 2006). Still, due to the rapid expansion of Wuhan city, both the price and demand for sand and gravel has increased, and as a result, a large number of sand mining boats have come to Poyang Lake. Larger ships leave and enter the lake at a rate of 2 ships/min (Zhang, 2007) and this number is likely even more today. Approximately 160 sand dredging enterprises are operating on the lake (Li, 2008). These dredging activities are mostly concentrated in the channel in the northern part of Poyang Lake. This area has the highest density of Yangtze finless porpoises (YFPs) (Yang et al., 2000). It has been assumed that the noise produced by the dredging and the heavy boat traffic has significantly changed the acoustic environment of Poyang Lake (Jing, 2008), resulting in acoustic pollution (Schelle, 2010). Furthermore, such high-density dredging projects have caused the death of local wildlife, including YFPs (Jing, 2008).

The YFP is a critically endangered (Mei et al., 2014) freshwater cetacean (Wang, 2009) endemic in the Yangtze River (lower and middle reaches) and its two adjoining lakes (Poyang and Dongting Lakes) (Gao and Zhou, 1993). A series of studies have reported a continuous reduction in the number of YFPs from more than 2,500 YFPs in 1991 (Zhang et al., 1993) to 1,225 (Zhao et al., 2008), 1,800 (Zhao et al., 2008), and recently in 2012, the total population was estimated to be 1,040 individuals (Mei et al., 2014). Of these 1,040 YFPs, ~400 individuals thrive in Poyang Lake (Zhao et al., 2008). In Poyang Lake, due to high turbidity and tremendous acoustic pollution, resulting from sand mining and heavy vessel traffic, the sonar system of YFPs is severely disturbed, resulting in navigational failure, difficulty in sensing prey and escaping danger, and migration failure between the Yangtze River and Poyang Lake (Wang et al., 2006; Zhang, 2007). In cetaceans, a series of studies have reported various organ injuries and sudden death because of collisions with the vessel and acoustic pollution (Freitas, 2004; Fernández et al., 2005; Cox et al., 2006). In addition, noise caused by heavy vessel traffic compromises nearly all the normal behaviors in cetaceans, including reproduction, energy expenditure, and energy acquisition (Hastie et al., 2003; Lusseau, 2003; Constantine et al., 2004; Lemon et al., 2006; Miller et al., 2008).

Noise is a potential stressor for all cetaceans (Wright et al., 2007). It stimulates the Hypothalamic-pituitary-adrenal (HPA) axis and it increases the level of cortisol secretion (Rolland et al., 2012). The higher the cortisol level the more the Hypothalamic-pituitary-gonadal (HPG) axis is suppressed (Bethea et al., 2008). Both acute and chronic stress resulting from acoustic pollution can increase the expression of RFamide-related peptides (RFRPs) in the hypothalamus and apoptosis of Leydig cells in the testes. Similarly, acoustic pollution inhibits the expression of Kisspeptin 1(KISS1) and Gonadotrophin-releasing-hormone (GnRH) in the hypothalamus. As a result, the secretion of pituitary Luteinizing Hormone (LH) and Follicle Stimulating Hormone (FSH) is reduced, which ultimately compromises steroidogenesis and gametogenesis (Nabi et al., 2018). Furthermore, acoustic pollution has negative effects on the physiology of the thyroid gland and this can cause metabolic disorders (Ramezani et al., 2014). Noise also has negative effects on the hematology and the biochemistry of cetaceans (St Aubin and Geraci, 1989; Asper et al., 1990; Bossart et al., 2001). Due to the presence of heavy vessel traffic in Poyang Lake, the objective of this study was to investigate the physiological effects of high vessel traffic on the hematology, biochemistry, and hormonal profile by comparing two different YFPs populations. One population living in Poyang Lake and the second living the Tian-E-Zhou Oxbow, where vessel traffic is negligible.

## Materials and methods

### Study location

In China, Poyang Lake, also named the Kingdom of rare birds, is the largest freshwater lake. It is located at longitude 115° 47′-116° 45′ east and latitude 28° 22′-29° 45′ north. The total area of the lake is 1.6 × 10^5^ km^2^. However, its size fluctuates with the season. It is fed by the Ganjiang River, Raohe River, Xiushui River, Xinjiang River, and Fuhe River. Finally, water empties into the Yangtze River (Jing, 2008; Sun et al., 2012; Dong, 2013; Vision Times Chinese., 2014). Poyang Lake is exposed to heavy vessel traffic and to chronic acoustic pollution arising from dynamite explosions, heavy vessel trafficking, and dredging (Wang, 2009; Schelle, 2010).

The Tian-E-Zhou Oxbow (E11 2°31′-112°36′, N29°46′-29°51′) is located near Shishou city in Hubei Province. It is an oxbow shaped semi-natural reserve formed in 1972 due to a deviation in the natural flow of the Yangtze River. The total length of the oxbow is about 21 km with a width of about 1–2 km (Hao et al., 2009; Wang, 2013). In the reserve, there is no dredging, or intensive shipping and even fishing is banned at certain times of the year. The reserve is managed regularly and the YFPs are assessed for health, fertility, and also investigated for research (Hao et al., 2009).

### Animal chasing, catching, handling, and release

In both populations, sound chase and net capture methods were used for animal capturing (Hua, 1987). A detailed explanation of the method is found in a paper by Hao et al. (2009). Each day, 5–10 YFPs in both populations were gently chased by several parallel fishing boats for approximately 15 min. The speed of fishing boats was slower than 10 km/h and the noise was 4.5 hp. The YFPs were allowed to swim in an open area where a spacious enclosure was made by using fishermen nets. The animals were confined to one section of the reserve overnight. The nets were enough soft to avoid injuries and allow fish to pass through it. The next morning, after gradually reducing the enclosed area, the animals were taken out of the water and transported to the medical boat for a physical examination and blood sampling. This process took ~15 min. The animals were put on a sponge mattress and were gently restrained. Each animal was sampled within 1 min after arrival. The methodology during the capture event, the blood sampling procedure and the timing of blood collection were consistent for both populations. There was a very little variation between the capture timing of both populations. During the entire process, behavioral reactions, breathing frequency, skin hydration, and the general health of all the animals were strictly monitored. All animals were then gently released back into their environment immediately after sampling.

### Study design

For the study, a total of 43 male YFPs were recruited from Poyang Lake (*n* = 20) and the Tian-E-Zhou Oxbow (*n* = 23). The YFPs in Tian-E-Zhou Oxbow were all adults. However, the YFPs in Poyang Lake were divided into juvenile males (*n* = 08) and adult males (*n* = 12) on the basis of body length (Gao and Zhou, 1993). In the Tian-E-Zhou Oxbow, the YFPs catching operation was carried out in the years 2002 (Winter/*n* = 06) and 2003 (Winter/*n* = 07). In Poyang Lake, the YFPs were captured during a physical examination project conducted in 2009 (Winter/*n* = 20).

### Blood sampling

Approximately 10 ml blood samples were aseptically obtained at a single event from the main vein on the dorsal side of the tail fluke, using a 10 ml heparinized syringe (Gemtier, G/Ø/ L: 21/0.7/31 mm, 201502, Shanghai, China). For hematology, ~2 ml of the blood collected was poured into heparinized tubes (Nihon, 161–8560, Tokyo, Japan). All the remaining blood samples were then transferred to a centrifuge tube (Corning, 14831, New York, America) for serum separation through centrifugation (Eppendorf AG, 22332, Hamburg, Germany) at 1,500 × g for 15 min. The serum was then immediately transferred to frost-free plastic tubes and stored in liquid nitrogen. In the laboratory, all samples were stored at −25°C. After blood sampling, standard body length and body mass were collected from each finless porpoise as soon as possible (American Society of Mammalogists, 1961).

### Complete blood count

Blood parameters such as; Hemoglobin (Hb), Red Blood Cells (RBCs), Hematocrit (HCT), Mean Corpuscular Volume (MCV), Mean Corpuscular Hemoglobin Concentration (MCHC), Mean Corpuscular Hemoglobin (MCH), Platelets (PLT), Eosinophils, Basophils, Monocytes, Lymphocytes, Neutrophils, and White Blood Cells (WBCs) were analyzed using a hematology analyzer (Beckman-Coulter, DxH 800, Porto, Portugal) according to the manufacturer's instructions.

### Biochemical analysis

The lipid profile; [Triglyceride (TG), Total Cholesterol (TC), Low Density Lipoprotein cholesterol (LDL-c), High Density Lipoprotein cholesterol (HDL-c)]; the liver function parameters [Gamma-glutamyl Transferase (GGT), Aspartate amino Transferase (AST), Alkaline Phosphatase (ALP), Alaline amino Transferase (ALT), Total Bile Acid (TBA), Direct Bilirubin (D-BILI), Indirect Bilirubin (I-BILI), Total Bilirubin (T-BILI)]; the enzymes; [Creatine Kinase (CK), Lactate Dehydrogenase (LDH), Amylase (AMS)]; Electrolytes (Na^+^, K^+^, Cl^−^, Ca^2+^, PO4^3−^, Mg^+2^, Fe^+2^); and other biochemical parameters, such as Globulin (GLB), Albumin (AlB), Total Protein (TP), Blood Urea Nitrogen (BUN), Uric Acid (UA), Creatinine (Cr), Glucose (GLU), and Carbon Dioxide (CO_2_) were measured using a calibrated automated clinical chemistry analyzer (Beckman-Coulter, AU5400, Porto, Portugal) according to the manufacturer's instructions.

### Radioimmunoassay of serum hormone

For the analysis of serum cortisol, testosterone, estradiol, and thyroid hormones (T4, T3, fT3, fT4), the commercially available RIA kits were used according to the manufacturer's instructions (Tian-jin Leeco Biotechnological and Medical Products Inc., China). These kits have been validated by Hao et al. (2007, 2009) for accuracy and parallelism. All the samples were assayed in duplicates by the same person. The intra- and inter-assay coefficients of variability for cortisol were < 5 and < 10%, testosterone 8.1 and 5.5%, and estradiol 9.2 and 7.3%. For the thyroid hormones (T4, T3, fT3, fT4) the intra- and inter-assay coefficients of variability were within 5 and 10%. The hormones (fT3, fT4, cortisol, testosterone) were analyzed in both populations. However, T3, T4, and estradiol were only analyzed in the Tian-E-Zhou Oxbow YFPs.

### Statistical analyses

The studied parameters in both populations, as well as, in juvenile males and adult males of Poyang Lake were compared by the non-parametric Mann Whitney *U*-test using Graph Pad Prism, version 5.01 *(*Graph Pad Software Inc., San Diego, CA, USA). The relationship between cortisol and other hormonal, hematological, and biochemical parameters in each population were analyzed using the Pearson correlation. A *P* < 0.05 indicated a statistically significant difference. All the data is presented as mean ± SEM, median, upper 95% CI, lower 95% CI and *P*-value.

### Limitations

The main limitations of our study were the limited quantity of blood samples as they were used for different molecular, biochemical, hormonal and hematological studies. Therefore, some hormones like T3 and T4 were not analyzed in the Tian-E-Zhou Oxbow populations. Furthermore, due to technical and ethical issues, sampling in different seasons was not done at Poyang Lake.

## Results

### Hematology

The complete blood profile of Poyang Lake YFPs showed a statistically significantly higher level of RBCs, HCT, MCV, WBCs, and Eosinophils compared to the Tian-E-Zhou Oxbow YFPs (Table 1). However, in the Tian-E-Zhou Oxbow YFPs, only MCH and MCHC were statistically significantly higher.

**Table 1:** Comparison of blood parameters between the Poyang Lake (*n* = 12) and Tian-E-Zhou Oxbow (*n* = 13) adult male YFPs (winter season).

**Parameters**	**Mean ± SEM**	**Upper 95% CI**	**Lower 95% CI**	**Median**	***P***
Rbc (10^12^/L)	^a^4.98 ± 0.03 ^b^5.30 ± 0.12	5.08 5.58	4.88 5.03	4.99 5.35	0.0271
Hb (g/L)	^a^176.0 ± 2.81 ^b^157.5 ± 14.60	183.8 190.1	168.2 125.0	176.0 173.0	0.2482
HCT (%)	^a^43.52 ± 0.52 ^b^47.19 ± 0.98	44.98 49.43	42.06 44.95	43.70 48.25	0.0216
MCH (pg)	^a^35.18 ± 0.49 ^b^32.35 ± 0.67	36.56 33.84	33.80 30.85	35.50 32.40	0.0117
MCHC (g/L)	^a^409.8 ± 4.42 ^b^363.7 ± 3.07	422.1 370.6	397.5 356.9	409.0 361.0	0.0011
MCV (fL)	^a^86.0 ± 1.35 ^b^89.87 ± 1.36	89.76 92.92	82.24 86.82	85.60 89.90	0.0394
PLT (10^9^/L)	^a^139.4 ± 7.17 ^b^120.7 ± 14.58	159.3 154.3	119.5 87.06	130.0 107.0	0.2188
WBCs (10^9^/L)	^a^4.62 ± 0.12 ^b^6.40 ± 0.41	4.95 7.32	4.28 5.47	4.80 6.10	0.0011
Neutrophil (%)	^a^58.76 ± 3.12 ^b^48.08 ± 3.53	67.43 55.86	50.09 40.31	55.50 51.0	0.0558
Lymphocyte (%)	^a^25.84 ± 2.37 ^b^30.58 ± 3.70	32.45 38.73	19.23 22.44	25.70 27.00	0.3176
Monocyte (%)	^a^0.50 ± 0.22 ^b^1.12 ± 0.26	1.12 1.71	–0.12 0.53	0.50 1.25	0.1087
Eosinophil (%)	^a^13.50 ± 1.73 ^b^19.38 ± 1.04	18.31 21.67	8.69 17.08	12.00 19.00	0.0066
Basophil (%)	^a^0.020 ± 0.02 ^b^0.04 ± 0.04	0.07 0.13	–0.03 –0.05	0.00 0.00	0.3190

a *Tian-E-Zhou Oxbow YFPs*.

b *Poyang Lake YFPs*.

### Biochemistry

The level of liver enzymes (AST, ALP), HDL-C/LDL-C, GLB, UA, Glucose, K^+^, and AMS were statistically significantly higher in porpoises living in Poyang Lake. On the other hand, in the Tian-E-Zhou Oxbow YFPs, we observed statistically significantly higher levels of the lipid parameters (TC, LDL-C), DBIL, ALB, ALB/GLB, CO_2_, Na^+^, and LDH (Tables 2, 3).

**Table 2:** Comparison of biochemical parameters between the Poyang Lake (*n* = 12) and Tian-E-Zhou Oxbow (*n* = 13) adult male YFPs (winter season).

**Parameters**	**Mean ± SEM**	**Upper 95% CI**	**Lower 95% CI**	**Mean**	***P***
ALT (U/L)	^a^31.40 ± 3.32 ^b^33.80 ± 2.73	40.63 39.98	22.17 27.62	31.00 32.50	0.3393
AST (U/L)	^a^181.0 ± 8.17 ^b^209.7 ± 7.52	203.7 226.7	158.3 192.7	174.0 206.0	0.0097
AST/ALT	^a^5.96 ± 0.54 ^b^6.45 ± 0.43	7.47 7.42	4.44 5.47	5.63 6.10	0.3393
ALP (U/L)	^a^72.0 ± 9.14 ^b^119.5 ± 19.26	97.39 163.1	46.61 75.93	62.0 134.0	0.0376
GGT (U/L)	^a^42.60 ± 5.68 ^b^45.30 ± 3.47	58.37 53.16	26.83 37.44	38.0 41.50	0.2499
TBIL (μmol/L)	^a^4.08 ± 0.22 ^b^3.77 ± 1.03	4.69 6.10	3.46 1.43	4.30 2.35	0.1221
DBIL (μmol/L)	^a^1.28 ± 0.03 ^b^0.27 ± 0.03	1.38 0.35	1.17 0.18	1.30 0.30	0.0013
IBIL (μmol/L)	^a^2.80 ± 0.20 ^b^3.51 ± 1.05	3.35 5.89	2.24 1.12	3.00 2.05	0.1770
TBA (μmol/L)	^a^7.72 ± 1.41 ^b^6.78 ± 1.88	11.65 11.04	3.79 2.52	9.30 4.80	0.1855
TC (mmol/L)	^a^6.23 ± 0.28 ^b^5.41 ± 0.22	7.02 5.91	5.44 4.91	6.39 5.36	0.0249
TG (mmol/L)	^a^1.10 ± 0.09 ^b^0.98 ± 0.12	1.38 1.27	0.83 0.70	1.05 0.98	0.2198
HDL-C (mmol/L)	^a^3.35 ± 0.17 ^b^3.02 ± 0.12	3.83 3.30	2.86 2.75	3.45 2.96	0.0888
LDL-C (mmol/L)	^a^1.60 ± 0.11 ^b^0.22 ± 0.02	1.92 0.27	1.27 0.18	1.65 0.23	0.0003
HDL-C LDL-C	^a^2.10 ± 0.05 ^b^14.0 ± 0.99	2.26 16.24	1.95 11.76	2.09 15.03	0.0013
TP (g/L)	^a^74.22 ± 0.75 ^b^75.37 ± 2.16	76.31 80.26	72.13 70.48	73.70 73.75	0.4296
ALB (g/L)	^a^64.38 ± 1.08 ^b^46.08 ± 0.91	67.38 48.14	61.38 44.02	62.90 45.30	0.0003
GLB (g/L)	^a^9.84 ± 0.76 ^b^29.16 ± 1.30	11.95 32.12	7.72 26.20	10.10 28.05	0.0013
ALB/GLB	^a^6.76 ± 0.68 ^b^1.59 ± 0.04	8.66 1.68	4.86 1.49	6.24 1.59	0.0013
BUN (mmol/L)	^a^15.82 ± 1.33 ^b^15.92 ± 1.17	19.54 18.58	12.11 13.25	17.32 14.89	0.4296
Urea (μmol/L)	^a^18.02 ± 1.77 ^b^45.03 ± 4.90	22.94 56.13	13.10 33.93	17.10 43.45	0.0003
CO_2_ (mmol/L)	^a^28.36 ± 1.26 ^b^19.26 ± 0.76	31.88 20.99	24.84 17.53	27.90 18.80	0.003
Glucose (mmol/L)	7.40 ± 0.21 8.09 ± 0.58	8.00 9.42	6.79 6.76	7.23 8.22	0.0276

a *Tian-E-Zhou Oxbow YFPs*.

b *Poyang Lake YFPs*.

**Table 3:** Comparison of electrolytes and enzymes between the Poyang Lake (*n* = 12) and Tian-E-Zhou Oxbow (*n* = 13) adult male YFPs (winter season).

**Parameters**	**Mean ± SEM**	**Upper 95% CI**	**Lower 95% CI**	**Median**	***P***
Cr (μmol/L)	^a^92.36 ± 6.65 ^b^80.50 ± 2.32	110.8 85.75	73.88 75.25	89.50 82.20	0.0823
K^+^ (mmol/L)	^a^3.19 ± 0.13 ^b^4.16 ± 0.10	3.55 4.41	2.83 3.91	3.24 4.09	0.0005
Na^+^ (mmol/L)	^a^155.4 ± 0.49 ^b^153.2 ± 0.77	156.8 155.0	154.0 151.4	155.4 153.8	0.0210
Cl^−^ (mmol/L)	^a^109.1 ± 0.96 ^b^110.0 ± 1.12	111.7 112.6	106.4 107.4	110.0 111.6	0.1928
Ca^2+^ (mmol/L)	^a^2.57 ± 0.03 ^b^2.62 ± 0.03	2.66 2.71	2.49 2.53	2.55 2.59	0.2738
PO4^3−^ (mmol/L)	^a^1.04 ± 0.07 ^b^1.03 ± 0.10	1.24 1.26	0.84 0.79	1.03 1.20	0.4734
Mg^2+^ (mmol/L)	^a^0.66 ± 0.02 ^b^0.75 ± 0.03	0.73 0.84	0.60 0.66	0.69 0.71	0.1148
Fe^2+^ (μmol/L)	^a^24.70 ± 1.11 ^b^30.63 ± 2.41	27.80 36.19	21.60 25.07	24.50 31.10	0.0734
CK (U/L)	^a^97.0 ± 24.04 ^b^104.9 ± 25.65	163.8 164.0	30.25 45.74	78.0 87.0	0.4734
LDH (U/L)	^a^220.8 ± 13.18 ^b^152.9 ± 4.93	257.4 164.1	184.2 141.7	229.0 148.5	0.0024
AMS (U/L)	^a^5.60 ± 0.40 ^b^9.50 ± 0.42	6.71 10.47	4.48 8.53	5.00 10.00	0.0023

a *Tian-E-Zhou Oxbow YFPs*.

b *Poyang Lake YFPs*.

### Serum hormones

In the adult YFPs living in Poyang Lake, serum cortisol, fT3, and fT4 were significantly higher. Only testosterone was significantly higher in the Tian-E-Zhou Oxbow adult YFPs (Table 4). Similarly, in Poyang Lake juvenile males, serum cortisol, fT3, and fT4 were significantly higher than in the adult males of Tian-E-Zhou Oxbow (Table 5). Testosterone, both in the adult males of Poyang Lake and Tian-E-Zhou Oxbow, was statistically significantly higher compared to the juvenile males of Poyang Lake (Tables 5, 6).

**Table 4:** Comparison of serum hormones between the adult males of Poyang Lake (*n* = 12) and Tian-E-Zhou Oxbow (*n* = 13) YFPs (winter season).

**Parameters**	**Mean ± SEM**	**Upper 95% CI**	**Lower 95%**	**Median**	***P*-value**
Cortisol (ng/mL)	^a^149.4 ± 11.54 ^b^856.6 ± 18.72	175.1 898.3	123.6 814.9	144 858.5	< 0.0001
Testosterone (ng/dL)	^a^122.5 ± 21.90 ^b^77.74 ± 1.60	170.7 81.28	74.32 74.20	101.8 79.06	0.0268
fT3 (pg/mL)	^a^19.47 ± 0.12 ^b^402.1 ± 41.58	21.75 493.6	11.20 310.6	16.43 402.3	< 0.0001
fT4 (pg/mL)	31.51 ± 5.42 261.0 ± 18.83	43.59 303.0	19.44 219.1	22.99 255.4	< 0.0001

a *Tian-E-Zhou Oxbow YFPs*.

b *Poyang Lake YFPs*.

**Table 5:** Comparisons of serum hormones between the adult males of Tian-E-Zhou Oxbow (*n* = 13) and juvenile males of Poyang Lake (*n* = 08) (winter season).

**Parameters**	**Mean ± SEM**	**Upper 95% CI**	**Lower 95%**	**Median**	***P*-value**
Cortisol (ng/mL)	^a^149.4 ± 11.54 ^b^874.6 ± 26.87	175.1 937.6	123.6 810.5	144 905.9	< 0.0001
Testosterone (ng/dL)	^a^122.5 ± 21.90 ^b^41.62 ± 8.01	170.7 60.58	74.32 22.67	101.8 37.52	0.0020
fT3 (pg/mL)	^a^19.47 ± 0.12 ^b^493.2 ± 55.21	21.75 623.7	11.20 362.6	16.43 561.3	< 0.0001
fT4 (pg/mL)	^a^31.51 ± 5.42 ^b^276.2 ± 14.03	43.59 309.3	19.44 243.0	22.99 269.3	< 0.0001

a *Tian-E-Zhou Oxbow adult YFPs*.

b *Poyang Lake juvenile YFPs*.

**Table 6:** Comparison of serum hormones between the adult (*n* = 12) and juvenile males (*n* = 08) of Poyang Lake YFPs (winter season).

**Parameters**	**Mean ± SEM**	**Upper 95% CI**	**Lower 95% CI**	**Median**	***P***
Cortisol (ng/mL)	^a^856.6 ± 18.72 ^b^874.0 ± 26.87	898.3 937.6	814.9 810.5	858.5 905.9	0.2949
Testosterone (ng/dL)	^a^77.74 ± 1.60 ^b^41.62 ± 8.01	81.28 60.58	74.20 22.67	79.06 37.52	< 0.0001
fT3 (pg/mL)	^a^402.1 ± 41.58 ^b^493.2 ± 55.21	493.6 623.7	310.6 362.6	402.3 561.3	0.0985
T3 (ng/mL)	^a^21.32 ± 3.86 ^b^20.33 ± 2.01	29.93 25.09	12.71 15.58	20.75 19.97	0.4209
fT4 (pg/mL)	^a^261.0 ± 18.83 ^b^276.2 ± 14.03	303.0 309.3	219.1 243.0	255.4 269.3	0.2782
T4 (μg/mL)	^a^4.28 ± 0.27 ^b^3.97 ± 0.25	4.89 4.57	3.67 3.36	3.97 3.76	0.2220

a *Adult YFPs*.

b *Juvenile YFPs*.

### Correlations of cortisol with hormones, biochemical, and blood cells

**Figure 1:**
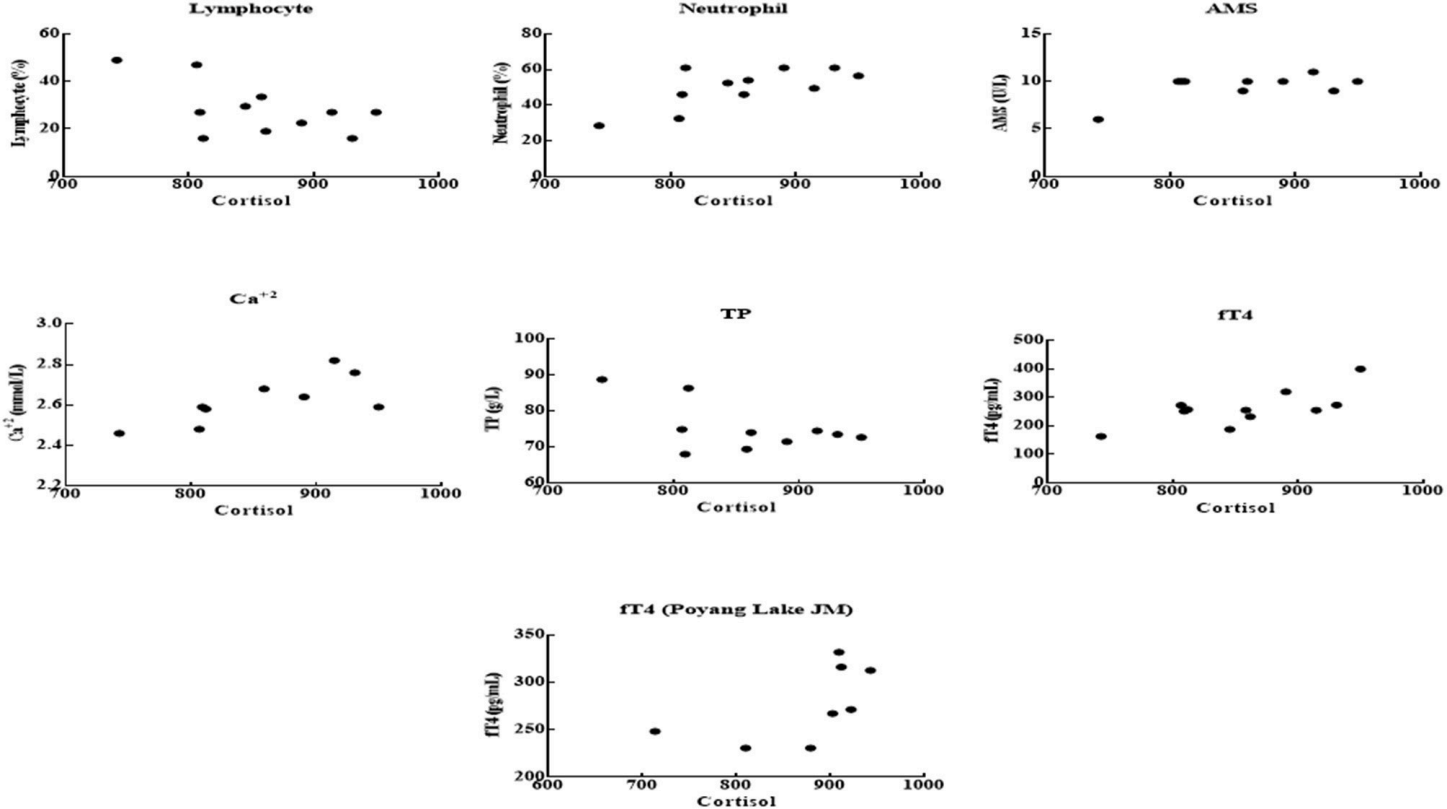
Correlations of the Poyang Lake JM (only cortisol vs. fT4) and AM between cortisol and blood, biochemical and hormonal parameters. Cortisol showed statistically significant negative associations with lymphocyte (*r* = −0.5851) and TP (*r* = −0.5565). The neutrophil (*r* = 0.6931), AMS (*r* = 0.5553), Ca^+2^ (*r* = 0.737), and fT4 (*r* = 0.7038) showed significant positive associations with cortisol. The juvenile male of Poyang Lake showed nearly a significant positive relationship between serum cortisol and fT4 (*r* = 0.5981; *P* = 0.0586).

The adult males of Poyang Lake showed a statistically significant positive correlation between cortisol and fT4 (*P* = 0.0078) (Figure 1). Similarly, we observed statistically significant positive correlations in AMS (*P* = 0.0478) and Ca^+2^ (*P* = 0.0117) and a statistically significant negative correlation in TP (*P* = 0.0474) with cortisol in Poyang Lake YFPs (Figure 1). Furthermore, neutrophils showed a statistically significantly (*P* = 0.0090) positive correlation and lymphocytes showed a statistically significant (*P* = 0.0293) negative correlation with serum cortisol in Poyang Lake YFPs (Figure 1).

### Body-weight/body-length (BW/BL) ratio

Comparison of the BW/BL between the adult males of both populations showed no significant difference. As expected, the BW/BL of the Poyang Lake JM was significantly lowered than the AM.

## Discussion

### Hematology

In porpoises living in Poyang Lake, we observed significantly higher levels of RBCs, HCT, MCV, WBCs, and eosinophils. The noise and heavy vessel traffic in Poyang Lake could have increased the diving activity of the animals. As a result, more oxygen is utilized. To facilitate oxygen transport, the concentration of HCT increases, which further encourages the synthesis of RBCs (Panneton, 2013; Fahlman et al., 2018). Furthermore, the significantly higher levels of WBCs and eosinophils in porpoises living in Poyang Lake could also be related to anthropogenic stress. Such an association has been observed in bottlenose dolphins (Asper et al., 1990), and beluga whales (St Aubin and Geraci, 1989). The statistically significant positive correlation observed in the Poyang Lake YFPs between the cortisol and neutrophil has been reported by Davis et al. (1991) in humans after exogenous intravenous administration of cortisol (Davis et al., 1991). Similarly, in the harbor seal (*Phoca vitulina*), exogenous administration of adrenocorticotrophic hormone (ACTH) increased neutrophil and decrease lymphocyte counts (Keogh and Atkinson, 2015).

### Biochemistry

In Poyang Lake YFPs, the significantly higher levels of serum glucose could be either due to stress, higher physical activities due to high vessel traffic, and/or hyperthyroidism (Bossart et al., 2001; Richter et al., 2001). Both the noise exposure and higher physical activity increases metabolic rate. More glucose is therefore needed to provide energy for elevated muscular activities (Richter et al., 2001; Castellini and Castellini, 2004). Higher physical activities increase muscle blood flow, glucose delivery, muscle membrane glucose transport capacity, muscle glycogen synthase activity, and other enzymatic activities associated with glucose metabolism (Richter et al., 2001). The intense physical activities produce varying amounts of stress depending on the intensity and duration. A statistically significant positive correlation in Poyang Lake YFPs between cortisol and AMS suggests that AMS can be used as a stress marker resulting from physical exertion (Allen, 2014). Similarly, the likely higher strenuous muscular activities of Poyang Lake YFPs in response to vessel trafficking also significantly increase serum K^+^ concentrations. The higher K^+^ concentration reflects leaking of the K^+^ into the bloodstream from hyperactive muscles (Geraci and Medway, 1973).

We observed higher TC and LDL-c in the Tian-E-Zhou Oxbow YFPs and higher HDL-c/LDL-c in Poyang Lake YFPs suggesting the effects of physical activities and/or food quality. However, we did not observe a statistically significant difference in the BW/BL between the two populations of adult males. Therefore, this is likely linked to the physical activities of the YFPs. This has been reported in captive and wild YFPs (Nabi et al., 2017), and beluga whales (*Delphinap terus leucas*) (St Aubin and Geraci, 1989; Cook et al., 1990).

Similarly, we found statistically significantly higher levels of AST and ALP in porpoises living in Poyang Lake compared to the statistically significantly higher levels of DBILI in the Tian-E-Zhou Oxbow YFPs. The statistically significantly higher serum AST and ALP in Poyang Lake YFPs could also be attributed to the persistent strenuous physical activities as reported by several studies (Bürger-Mendonça et al., 2008; Ghorbani and Gaeini, 2013; Nazari et al., 2014; Ekun et al., 2017). The enzymes AST and ALP are present both in the muscles and the liver. During strenuous physical activities, damage to muscle tissue can occur, which allows these enzymes to enter the bloodstream. To provide a continuous energy flow to the exercising muscles, AST catabolizes amino acids to produce Adenosine Triphosphate (ATP). Similarly, a higher serum ALP concentration indicates lipid peroxidation and gluconeogenesis, which are used for energy hemostasis (Nazari et al., 2014). Furthermore, the high concentration of thyroid hormones and high metabolism of Poyang Lake YFPs can also possibly cause oxidative damage to the hepatocytes thereby increasing the serum ALP and AST concentrations (Khemichian and Fong, 2011).

The significantly higher levels of serum GLB and AMS in Poyang Lake YFPs might be due to acoustic pollution induced stress (Haider et al., 1977; Petrakova et al., 2015). The negative correlation of cortisol with TP in Poyang Lake population could possibly be due to glucocorticoids-induced increase in protein degradation and decrease protein synthesis (Kuo et al., 2013; Corazza et al., 2014). Similarly, the positive correlation of cortisol with Ca^+2^ has been reported in human (Radhi, 2014). In a variety of vertebrates, serum Ca^+2^ concentration increases in response to intense physical activities (Ruben and Bennett, 1981). As parathyroid gland regulates Ca^+2^ hemostasis, therefore further studies in YFPs are needed to investigate if parathyroid hormone can be used as a biomarker for physical stress.

### Serum hormones

We observed statistically significantly higher cortisol level in the heavily trafficked and noisy Poyang Lake YFPs vs. the control Tian-E-Zhou Oxbow YFPs. This difference might reflect differences in baseline cortisol levels, stress response during handling, or the additive effects of both. In wild cetaceans, the process of animals handling, and blood collection induces stress. Therefore, it is difficult to investigate the actual physiological response of an animal to environmental stressor in the wild (Fair et al., 2014; Atkinson et al., 2015). Furthermore, our single observatory data is not enough to indicate baseline values, especially for stress-related parameters. Therefore, it is essential to consider the possible combined effects of environment and capture/handling responses in the interpretation of results related to hormones that are secreted immediately (Fair et al., 2017).

The higher cortisol level in the heavily trafficked Poyang Lake population might be in response to acoustic pollution as compared to the control Tian-E-Zhou Oxbow population. The higher cortisol in Poyang Lake YFPs might be an adaptation for maintaining the physiological integrity and ultimately the survival of an organism in the presence of potential stressors (Ulrich-Lai and Herman, 2009; Sheriff et al., 2011). For all cetaceans, noise is a stressor regardless of age (Wright et al., 2007). Several studies on North Atlantic right whales (*Eubalaena glacialis*), and bottlenose dolphins (*Tursiops truncates)* reported a higher level of cortisol in response to the noise emitted either from ships or from water seismic guns (Romano et al., 2004; Rolland et al., 2012). There is a direct correlation between noise and fecal glucocorticoid levels. With increasing ship noise, there is increasing levels of fecal glucocorticoids (Rolland et al., 2012). This happens through a generalized stress response, such as the elevation of glucocorticoid levels (cortisol and corticosterone) by HPA-axis activation (St Aubin and Geraci, 1989, 1990; St Aubin et al., 1996). An increased heart rate was detected in captive bottlenose dolphins in response to threatening sounds. The cardiac response patterns were consistent with the physiological defense and startle responses in terrestrial mammals and birds (Miksis et al., 2001). Repeated and extended exposure to noise can cause a variety of physiological issues, including suppression of reproduction (Wright et al., 2007). The statistically significantly higher cortisol, lowered serum testosterone level, and fertility rates in Poyang Lake YFPs compared to Tian-E-Zhou Oxbow YFPs (Wang, 2015), suggests the negative effect of anthropogenic noise on reproduction. Cortisol affects the HPG-axis at many levels; at the hypothalamic level (by decreasing the synthesis and secretion of gonadotropin-releasing hormone), at the pituitary level (by decreasing the synthesis and secretion of LH and FSH) and at the gonadal level (affecting steroidogenesis and/or gametogenesis) (Whirledge and Cidlowski, 2010). Several studies have reported the negative effects of noise and vibration on the reproductive physiology of mammals directly or indirectly (Algers et al., 1978; Penkov and Tzyetkov, 1999; Rabin et al., 2003; Nabi et al., 2014). The statistically significantly lower serum testosterone levels in juvenile male porpoises living in Poyang Lake has been previously reported in captive and wild YFPs, respectively (Daoquan et al., 2006; Hao et al., 2007).

Glucocorticoids can alter animal behavior depending upon the environmental context. It includes abandoning an area and fleeing the stressor (Fair et al., 2017). Therefore, cetaceans exposed to heavy vessel traffic have a higher metabolic rate in response to altered diving behavior (Peng et al., 2015). The elevated thyroid hormones promote the hypermetabolic state characterized by increased resting energy expenditure, lipolysis, and gluconeogenesis (Mullur et al., 2014). In our study, we observed statistically significantly higher serum levels of fT3 and fT4 in the Poyang Lake YFPs. Like our study, St Aubin et al. (1996) and Ortiz et al. (2000) also found significantly higher levels of fT3 and fT4 in wild manatees (*Trichechus manatus*) compared to captive manatees. A similar positive relationship between physical activity and serum T4 and fT4 in healthy young individuals has been reported (Licata et al., 1984; Figen et al., 2005). Captivity in cetaceans and manatees limit the physical activity of animals causing a reduction in thyroid hormone concentrations compared to its free-ranging counterparts (St Aubin and Geraci, 1988; Ortiz et al., 2000). The statistically significantly higher concentration of fT3 and fT4 and the positive correlation between cortisol and fT4 in the Poyang Lake YFPs suggest increased physical activity resulting from the tremendous anthropogenic activities as indicated by statistically significantly higher cortisol levels (St Aubin and Geraci, 1988). The association between higher cortisol levels and higher levels of fT3 and fT4 has been reported in wild bottlenose dolphins (St Aubin et al., 1996). In many species, including captive bottlenose dolphins (Ortiz et al., 2000), beluga whales (*Delphinapterus leucas*) (St Aubin and Geraci, 1988), and reindeer (*Rangifer tarandus tarandus*) (Ringberg et al., 1978), reduced thyroid hormones levels are associated with lower physical activity induced by captivity. Although, several studies in cetaceans have documented reports about circulating concentrations of TH (Greenwood and Barlow, 1979; Orlov et al., 1988; St Aubin, 2001; Debier et al., 2005; Fair et al., 2011), still, limited studies are available on the thyroid system of odontocetes (porpoises, dolphins, and toothed whales) living in the wild and the association with anthropogenic activities.

## Conclusions and future recommendations

In summary, we found statistically significantly increased stimulatory activity of the adrenal and thyroid gland in Poyang Lake population compared to Tian-E-Zhou Oxbow. In response to stress, hormones (especially fT4), blood cell counts (neutrophils and lymphocytes), and several biochemical parameters are affected in the Poyang Lake population. The cortisol levels of YFPs living in Poyang Lake did not show a statistically significant correlation with testosterone. The overall statistically significantly lower level of serum testosterone and variations in blood, biochemical, and other hormonal parameters compared to Tian-E-Zhou Oxbow YFPs suggests the possible effects of heavy vessel traffic and dredging. Alterations in the hormonal, hematological, and biochemical parameters will have serious consequences related to metabolism, immunity, reproduction, and general well-being. Unfortunately, the number of vessels, dredging, and other anthropogenic activities in Poyang Lake are increasing from year to year and therefore this problem requires serious attention as it affects the conservation of YFPs. A ban on sand dredging, especially in YFPs rich areas, regulating the number of vessels, and the speed of the vessels is essential. Especially, during the breeding season, dredging and vessels traffic should be banned or minimized. This is because compromising the reproduction of a critically endangered species for a long period can easily cause extinction. Introduction of quiet ships and assigning special routes could possibly minimize the impact. Economic precedence always trumps environmental and biodiversity issues. Therefore, it is better to have more semi-natural reserves like the Tina-E-Zhou Oxbow where YFP's fertility can be increased by controlling anthropogenic activities. Furthermore, controlled experiments are needed to investigate the response of the HPG and HPA-axis toward underwater sounds at different intensities and identify the threshold frequency that stimulates the HPA and suppress the HPG-axis. Additionally, data regarding vessel traffic, acoustic pollutions, level of YFPs activities and reference levels for various physiological indices are needed for both populations.

## Ethics statement

The study was ethically reviewed and approved by the Ministry of Agriculture of the People's Republic of China and the Research Ethics Committee of Institute of Hydrobiology, Chinese Academy of Science. In this study, no surgical interventions, including euthanasia and anesthesia was used. The entire study strictly followed the Chinese law and ethical guidelines for wildlife.

## Author contributions

GN conceived the study, analyzed the data, and drafted the article. YH and DW collected the data. RM helped in writing the manuscript and critically reviewed the article. All authors read and approved the final manuscript.

### Conflict of interest statement

The authors declare that the research was conducted in the absence of any commercial or financial relationships that could be construed as a potential conflict of interest.

## Abbreviations

ATP: Adenosine Triphosphate
ALT: Alaline amino Transferase
AlB: Albumin
ALP: Alkaline Phosphatase
AMS: Amylase
AST: Aspartate amino Transferase
BUN, Blood Urea Nitrogen, CO_2_: Carbon Dioxide
CK, Creatine Kinase, Cr: Creatinine
DBILI: Direct Bilirubin
GGT: Gamma-glutamyl Transferase
GLB: Globulin
GLU: Glucose
HCT: Hematocrit
Hb: Hemoglobin
HDL-c: High Density Lipoprotein cholesterol
HPA: Hypothalamic-Pituitary-Adrenal
HPG: Hypothalamic-Pituitary-Gonadal
IBILI: Indirect Bilirubin
LDH: Lactate Dehydrogenase
LDL-c: Light Density Lipoprotein cholesterol
MCH: Mean Corpuscular Hemoglobin
MCHC: Mean Corpuscular Hemoglobin Concentration
MCV: Mean Corpuscular Volume
PLT: Platelets
RBCs: Red Blood Cells
TBA: Total Bile Acid
TBILI: Total Bilirubin
TC: Total Cholesterol
TP: Total Protein
TG: Triglyceride
UA: Uric Acid
WBCs: White Blood Cells
YFPs: Yangtze Finless Porpoises.

## References

[B1] Algers B. Ekesbo I. Stromberg S. (1978). The impact of continuous noise on animal health. Acta Vet. Scand. 67, 1–26. 356566

[B2] Allen S. (2014). Salivary Alpha-Amylase as an Indicator of Body Stress Following an Acute Session of Repetitive Jumping. UNLV Theses, Dissertations, Professional Papers, and Capstones. 2055. Available online at: https://digitalscholarship.unlv.edu/thesesdissertations/2055

[B3] American Society of Mammalogists (1961). Standardized methods for measuring and recording data on the smaller cetaceans. J. Mamm. 42, 471–6. 10.2307/1377364

[B4] Asper E. D. Cornell L. H. Duffield D. A. Odell D. K. Joseph B. E. Stark B. I. (1990). Haematology and serum chemistry values in bottlenose dolphins, in The Bottlenose Dolphin, eds Leatherwood S. Reeves R. R. (San Diego, CA: Academic Press), 478–485.

[B5] Atkinson S. Crocker D. Houser D. Mashburn K. (2015). Stress physiology in marine mammals: how well do they fit the terrestrial model? J. Comp. Physiol. B 185, 463–486. 10.1007/s00360-015-0901-0 25913694

[B6] Bethea C. L. Centeno M. L. Cameron J. L. (2008). Neurobiology of stress-induced reproductive dysfunction in female macaques. Mol. Neurobiol. 38, 199–230. 10.1007/s12035-008-8042-z 18931961 PMC3266127

[B7] Bossart G. D. Reidarson T. H. Dierauf L. A. Duffield D. A. (2001). Clinical pathology, in Marine Mammal Medicine, eds Dierauf L.A. Gulland F. M. D. (Boca Raton, FL: CRC Press Inc), 383–436.

[B8] Bürger-Mendonça M. Bielavsky M. Barbosa F. C. (2008). Liver overload in Brazilian triathletes after half-Ironman competition is related muscle fatigue. Ann. Hepatol. 7, 245–248. 18753992

[B9] Castellini M. A. Castellini J. M. (2004). Defining the limits of diving biochemistry in marine mammals. Comp. Biochem. Physiol. B Biochem. Mol. Biol. 139, 509–518. 10.1016/j.cbpc.2004.09.011 15544972

[B10] Constantine R. Brunton D. H. Dennis T. (2004). Dolphin-watching tour boats change bottlenose dolphin (*Tursiops truncatus*) behaviour. Biol. Conserv. 117, 299–307. 10.1016/j.biocon.2003.12.009

[B11] Cook R. A. Michael K. S. Ellen S. D. (1990). Circulating levels of vitamin E, cholesterol, and selected minerals in captive and wild Beluga Whales (*Delphinapterus leucas*). J. Zoo Wildl. Med. 21, 65–69.

[B12] Corazza D. I. Sebastião É. Pedroso R. V. Andreatto C. A. A. Coelho M. G. M. Gobbi S. (2014). Influence of chronic exercise on serum cortisol levels in older adults. Eur. Rev. Aging Phys. Act. 11, 25–34. 10.1007/s11556-013-0126-8

[B13] Cox T. M. Ragen T. J. Read A. J. Vos E. Baird R. W. Balcomb K. (2006). Understanding the impacts of anthropogenic sound on beaked whales. J. Cetacean Res. Manage. 7, 177–187.

[B14] Daoquan C. Yujiang H. Qingzhong Z. Ding W. (2006). Reproductive seasonality and maturity of male *Neophocaena phocaenoides* asiaeorientalis in captivity: a case study based on the hormone evidence. Mar. Freshw. Behav. Phy. 39, 163–173. 10.1080/10236240600563396

[B15] Davis J. M. Albert J. D. Tracy K. J. Calvano S. E. Lowry S. F. Shires G. T.. (1991). Increased neutrophil mobilization and decreased chemotaxis during cortisol and epinephrine infusions. J. Trauma 31, 725–731. 10.1097/00005373-199106000-00001 2056538

[B16] Debier C. Ylitalo G. M. Weise M. Gulland F. Costa D. P. Le Boeuf B. J.. (2005). PCBs and DDT in the serum of juvenile California sealions: associations with vitamins A and E and thyroid hormones. Environ. Pollut. 134, 323–332. 10.1016/j.envpol.2004.07.012 15589659

[B17] Dong Y. (ed.). (2013). Background information of Poyang Lake and Yangtze finless porpoises, in Contingent Valuation of Yangtze Finless Porpoises in Poyang Lake, China (Dordrecht: Springer), 5–36.

[B18] Ekun O. A. Emiabata A. F. Abiodun O. C. Ogidi N. O. Adefolaju F. O. Ekun O. O. (2017). Effects of football sporting activity on renal and liver functions among young undergraduate students of a Nigerian tertiary institution. BMJ Open Sport Exerc. Med. 3:e000223. 10.1136/bmjsem-2017-000223 28761709 PMC5530113

[B19] Fahlman A. McHugh K. Allen J. Barleycorn A. Allen A. Sweeney J.. (2018). Resting metabolic rate and lung function in wild offshore common bottlenose dolphins, *Tursiops truncatus*, near Bermuda. Front. Physiol. 9:886. 10.3389/fphys.2018.00886 30065656 PMC6056772

[B20] Fair P. A. Montie E. Balthis L. Reif J. S. Bossart J. S. (2011). Influences of biological variables and geographic location on circulating concentrations of thyroid hormones in wild bottlenose dolphins (*Tursiops truncatus*). Gen. Comp. Endocrinol. 174, 184–194. 10.1016/j.ygcen.2011.08.021 21930130

[B21] Fair P. A. Schaefer A. M. Houser D. S. Bossart G. D. Romano T. A. Champagne C. D.. (2017). The environment as a driver of immune and endocrine responses in dolphins (*Tursiops truncatus*). PLoS ONE 12:e0176202. 10.1371/journal.pone.0176202 28467830 PMC5415355

[B22] Fair P. A. Schaefer A. M. Romano T. Bossart G. D. Lamb S. V. Reif J. S. (2014). Stress response of wild bottlenose dolphins (*Tursiops truncatus*) during capture-release health assessment studies. Gen. Comp. Endocrinol. 206, 203–212. 10.1016/j.ygcen.2014.07.002 25019655

[B23] Fernández A. Edwards J. F. Rodriguez F. Espinosa de los Monteros A. Herraez P. Castro P.. (2005). Gas and fat embolic syndrome' involving a mass stranding of beaked whales (family Ziphiidae) exposed to anthropogenic sonar signals. Vet. Pathol. 42, 446–457. 10.1354/vp.42-4-446 16006604

[B24] Figen C. Ismail P. Aysel P. Kursat K. Nevin I. Ozcan S. (2005). Exercise intensity and its effects on thyroid hormones. Neuroendocrinol. Lett. 26, 830–834. 16380698

[B25] Freitas L. (2004). The stranding of three Cuvier's beaked whales *Ziphius caviostris* in Madeira archipelago – May 2000. ECS Newslett. 42, 28–32.

[B26] Fu B. Wu B. Lue Y. Xu Z. Cao J. Niu D. (2010). Three Gorges Project: efforts and challenges for the environment. Prog. Phys. Geogr. 34, 741–754. 10.1177/0309133310370286

[B27] Gao A. Zhou K. (1993). Growth and reproduction of three populations of finless porpoise. *Neophocaena phocaenoides*. Chinese waters. Aquat Mamm. 19, 3–12.

[B28] Geraci J. R. Medway W. (1973). Simulated field blood studies in the bottle-nosed dolphin *Tursiops truncatus*. 2. Effects of stress on some hematologic and plasma chemical parameters. J. Wildl. Dis. 9, 29–33. 10.7589/0090-3558-9.1.29 4694586

[B29] Ghorbani P. Gaeini A. A. (2013). The effect of one bout high intensity interval training on liver enzymes level in elite soccer players. J. Basic Appl. Sci. 5, 1191–1194.

[B30] Greenwood A. G. Barlow C. E. (1979). Thyroid function in dolphins: radioimmunoassay measurement of thyroid hormones. Br. Vet. J. 135, 96–102. 10.1016/S0007-1935(17)32994-9 761064

[B31] Haider M. Kanz G. Koller M. Schmid H. (1977). Stress-induced blood protein and blood lipid changes and their dependence on learning and conditioning. Wien Klin. Wochenschr. 89, 18–23. 835282

[B32] Hao Y. J. Chen D. Q. Zhao Q. Z. Wang D. (2007). Serum concentrations of gonadotropins and steroid hormones of *Neophocaena phocaenoides* asiaeorientalis in middle and lower regions of the Yangtze River. Theriogenology 67, 673–680. 10.1016/j.theriogenology.2006.06.014 17196248

[B33] Hao Y. J. Zhao Q. Z. Wu H. P. Chen D. Q. Gong C. Li L. (2009). Physiological responses to capture and handling of free-ranging male Yangtze finless porpoises (*Neophocaena phocaenoides* asiaeorientalis). Mar. Freshw. Behav. Phy. 42, 315–327. 10.1080/10236240903302161

[B34] Hastie G. D. Wilson B. Tufft L. H. Thompson P. M. (2003). Bottlenose dolphins increase breathing synchrony in response to boat traffic. Mar. Mamm. Sci. 19, 74–84. 10.1111/j.1748-7692.2003.tb01093.x

[B35] Hua Y. Y. (1987). Live capture of the Chinese river dolphin Lipotes by the noise of small boats and the seine. Acta Hydrobiol. Sin. 11, 99–100.

[B36] Jing X. (2008). Change Detection of Hydro-Acoustic Environment for Yangtze Finless Porpoise Using Remote Sensing in Poyang Lake. Master thesis, International Institute for Geo-information Science and Earth Observation, Netherland; and Wuhan University, China 1–44.

[B37] Keogh M. J. Atkinson S. (2015). Endocrine and immunological responses to adrenocorticotrophic hormone (ACTH) administration in juvenile harbor seals (*Phoca vitulina*) during winter and summer. Comp. Biochem. Physiol. A Mol. Integr. Physiol. 188, 22–31. 10.1016/j.cbpa.2015.06.011 26086360

[B38] Khemichian S. Fong T. L. (2011). Hepatic dysfunction in hyperthyroidism. Gastroenterol. Hepatol. 7, 337–339. 21857837 PMC3127041

[B39] Kuo T. Harris C. A. Wang J. C. (2013). Metabolic functions of glucocorticoid receptor in skeletal muscle. Mol. Cell Endocrinol. 380, 79–88. 10.1016/j.mce.2013.03.003 23523565 PMC4893778

[B40] Lemon M. Lynch T. P. Cato D. H. Harcourt R. G. (2006). Response of travelling bottlenose dolphins (*Tursiops aduncus*) to experimental approaches by a powerboat in Jervis Bay, New South Wales, Australia. Biol. Conserv. 127, 363–372. 10.1016/j.biocon.2005.08.016

[B41] Li T. (2008). Crisis at Poyang Lake. China Dialogue 28th March. Available online at: http://www.chinadialogue.net/homepage/show/single/en/1846-Crisis-at-Poyang-Lake (Accessed April 21, 2018).

[B42] Licata G. Scaglione R. Novo S. Dichiara M. A. Di Vincenzo D. (1984). Behaviour of serum T3, rT3, TT4, FT4 and TSH levels after exercise on a bicycle ergometer in healthy euthyroid male young subjects. Boll. Soc. Ital. Biol. Sper. 60, 753–759. 6732949

[B43] Liu R. Wang D. Zhou K. (2000). Effects of water development on river cetaceans in China, in Biology and Conservation of Freshwater Cetaceans in Asia, eds Reeves R. R. Smith B. D. Kasuya T. (Gland; Cambridge: IUCNIUCN), 40–42.

[B44] Lixin Z. (2018). Bigger Ships to Drive Yangtze Growth. China Daily. Available online at: http://www.chinadaily.com.cn/a/201801/06/WS5a500344a31008cf16da5649.html (Accessed April 20, 2018).

[B45] Lusseau D. (2003). Male and female bottlenose dolphins *Tursiops* spp have different strategies to avoid interactions with tour boats in Doubtful Sound, New Zealand. Mar. Ecol. Prog. Ser. 257, 267–274. 10.3354/meps257267

[B46] Mei Z. Zhang X. Huang S. L. Zhao X. Hao Y. Zhang L. (2014). The Yangtze finless porpoise: on an accelerating path to extinction? Biol. Conserv. 172, 117–123. 10.1016/j.biocon.2014.02.033

[B47] Miksis J. L. Grund M. D. Nowacek D. P. Solow A. R. Connor R. C. Tyack P. L. (2001). Cardiac responses to acoustic playback experiments in the captive bottlenose dolphin (*Tursiops truncatus*). J. Comp. Psychol. 115, 227–232. 10.1037/0735-7036.115.3.227 11594491

[B48] Miller L. J. Solangi M. Kuczaj S. A. (2008). Immediate response of Atlantic bottlenose dolphins to high-speed personal watercraft in the Mississippi Sound. J. Mar. Biol. Assoc. 88, 1139–1143. 10.1017/S0025315408000908

[B49] Mullur R. Liu Y. Y. Brent G. A. (2014). Thyroid hormone regulation of metabolism. Physiol. Rev. 94, 355–382. 10.1152/physrev.00030.2013 24692351 PMC4044302

[B50] Nabi G. Amin M. Khan A. A. (2014). Reproductive health in rickshaw drivers: occupational exposure to environmental stressor. Bali. Med. J. 3, 78–84. 10.15562/bmj.v3i2.79

[B51] Nabi G. Hao Y. Zeng X. Wang D. (2017). Assessment of Yangtze finless porpoises (*Neophocaena asiaorientalis*) through biochemical and hematological parameters. Zool. Stud. 56:31 10.6620/ZS.2017.56-31 PMC6517755 31966230

[B52] Nabi G. McLaughlin R. W. Hao Y. Wang K. Zeng X. Khan S. (2018). The possible effects of anthropogenic acoustic pollution on marine mammals' reproduction: an emerging threat to animal extinction. Environ. Sci. Pollut. Res. Int. 1, 1–8. 10.1007/s11356-018-2208-7 29804251

[B53] Nazari Y. Mohamadimofrad A. Nazari A. Jamshidi R. Asjodi F. (2014). Response of liver enzymes to acute aerobic exercise in sedentary human subjects. N. Y. Sci. J. 7, 89–92.

[B54] Orlov M. V. Mukhyla A. M. Kulikov N. A. (1988). Hormonal indices in the bottle-nosed dolphin *Tursiops truncatus* in the norm and the dynamics of experimental stress. Sov. J. Evol. Biochem. Physiol. A 125, 317–324.

[B55] Ortiz R. M. MacKenzie D. S. Worthy G. A. J. (2000). Thyroid hormone concentrations in captive and free-ranging west Indian manatees (*Trichechus manatus*). J. Exp. Biol. 203, 3631–3637. 11060224 10.1242/jeb.203.23.3631

[B56] Panneton W. M. (2013). The mammalian diving response: an enigmatic reflex to preserve life? Physiology 28, 284–297. 10.1152/physiol.00020.2013 23997188 PMC3768097

[B57] Peng C. Zhao X. Liu G. (2015). Noise in the sea and its impacts on marine organisms. Hawkins WE. Int. J. Environ. Res. Public Health 12, 12304–12323. 10.3390/ijerph121012304 26437424 PMC4626970

[B58] Penkov A. Tzyetkov D. (1999). Effect of vibrations on male reproductive system and function. Cent. Eur. J. Public Health 7, 149–154. 10499149

[B59] Petrakova L. Doering B. K. Vits S. Engler H. Rief W. Schedlowski M.. (2015). Psychosocial stress increases salivary alpha-amylase activity independently from plasma noradrenaline levels. PLoS ONE 10:e0134561. 10.1371/journal.pone.0134561 26247781 PMC4527714

[B60] Rabin L. A. Mccowan B. Hooper S. L. Owings D. H. (2003). Anthropogenic noise and its effect on animal communication: an interface between comparative psychology and conservation biology. Int. J. Comp. Psychol. 16, 172–192.

[B61] Radhi H. (2014). Acute stress, salivary cortisol and calcium ions, in patients undergoing dental extraction procedure. MDJ 11, 111–121.

[B62] Ramezani N. Ahmadi R. Akbari S. H. Mohammadi S. (2014). Effects of noise pollution on thyroid function in rat, in International Conference on Earth, Environment and Life sciences. Available online at: http://iicbe.org/upload/4512C1214114.pdf (Accessed April 17, 2018).

[B63] Richter E. A. Derave W. Wojtaszewski J. F. P. (2001). Glucose, exercise and insulin: emerging concepts. J. Physiol. 535, 313–322. 10.1111/j.1469-7793.2001.t01-2-00313.x 11533125 PMC2278791

[B64] Ringberg T. Jacobsen E. Ryg M. Krog J. (1978). Seasonal changes in levels of growth hormone, somatomedin and thyroxine in free-ranging, semi-domesticated Norwegian reindeer [*Rangifer tarandus tarandus* (L.)]. Comp. Biochem. Physiol. 60, 123–126. 10.1016/0300-9629(78)90215-3

[B65] Rolland R. M. Parks S. E. Hunt K. E. Castellote M. Corkeron P. J. Nowacek D. P.. (2012). Evidence that ship noise increases stress in right whales. Proc. Biol. Sci. 279, 2363–2368. 10.1098/rspb.2011.2429 22319129 PMC3350670

[B66] Romano T. A. Keogh M. J. Kelly C. Feng P. Berk L. Schlundt C. E. (2004). Anthropogenic sound and marine mammal health: measures of the nervous and immune systems before and after intense sound exposure. Can. J. Fish Aquat. Sci. 61, 1124–1134. 10.1139/f04-055

[B67] Ruben J. A. Bennett A. F. (1981). Intense exercise, bone structure and blood calcium levels in vertebrates. Nature 291, 411–413. 10.1038/291411a0 7242662

[B68] Schelle P. (2010). River Dolphins & People: Shared Rivers, Shared Future. WWF International.

[B69] Sheriff M. J. Dantzer B. Delehanty B. Palme R. Boonstra R. (2011). Measuring stress in wildlife: techniques for quantifying glucocorticoids. Oecologia 166, 869–887. 10.1007/s00442-011-1943-y 21344254

[B70] Smith B. D. Reeves R. R. (2000). Report of the workshop on the effects of water development on river cetaceans, in Biology and Conservation of Freshwater Cetaceans in Asia, eds Reeves R. R. Smith B. D. Kasuya T. (Gland; Cambridge: IUCN), 26–28.

[B71] St Aubin DJ. (2001). Endocrinology, in CRC Handbook of Marine Mammal Medicine, eds Dierauf L. A. Gulland F. (Boca Raton, FL: CRC Press), 165–192.

[B72] St Aubin D. J. Geraci J. R. (1988). Capture and handling stress suppresses circulating levels of thyroxine (T4) and triiodothyronine (T3) in beluga whales *Delphinapterus leucas*. Physiol. Zool. 61, 170–175. 10.1086/physzool.61.2.30156148

[B73] St Aubin D. J. Geraci J. R. (1989). Adaptive changes in haematologic and plasma chemical constituents in captive beluga whales, *Delphinapterus leucas*. Can. J. Fish Aquat. Sci. 46, 796–803. 10.1139/f89-099

[B74] St Aubin D. J. Geraci J. R. (1990). Adrenal responsiveness to stimulation by adrenocorticotropic hormone (ACTH) in captive beluga whales, *Delphinapterus leucas*. Can. B Fish Aquat. Sci. 224, 149–157.

[B75] St Aubin D. J. Ridgway S. H. Wells R. S. Rhinehart H. (1996). Dolphin thyroid and adrenal hormones: circulating levels in wild and semidomesticated *Tursiops truncatus*, and influence of sex, age, and season. Mar. Mamm. Sci. 12, 1–13. 10.1111/j.1748-7692.1996.tb00301.x

[B76] Sun S. L. Chen H. S. Ju W. M. Song J. Li J. J. Ren Y. J. (2012). Past and future changes of stream flow in Poyang Lake Basin, Southeastern China. Hydrol. Earth Syst. Sci. 16, 2005–2020. 10.5194/hess-16-2005-2012

[B77] Turvey S. T. Pitman R. L. Taylor B. L. Barlow J. Akamatsu T. Barrett L. A.. (2007). First human-caused extinction of a cetacean species? Biol. Lett. 3, 537–540. 10.1098/rsbl.2007.0292 17686754 PMC2391192

[B78] Ulrich-Lai Y. M. Herman J. P. (2009). Neural regulation of endocrine and autonomic stress responses. Nat. Rev. Neurosci. 10, 397–409. 10.1038/nrn2647 19469025 PMC4240627

[B79] Vision Times Chinese (2014). Poyang Lake: China's Bermuda Triangle. Available online at: http://www.visiontimes.com/2014/01/12/poyang-lake-chinas-bermuda-triangle.html (Accessed April 15, 2016).

[B80] Wang D. (2009). Population status, threats and conservation of the Yangtze finless porpoise. Sci. Bull. 54, 3473–3484. 10.1007/s11434-009-0522-7

[B81] Wang D. (2013). Population status, threats and conservation of the Yangtze finless porpoise. Sci. Bull. 27, 46–55.

[B82] Wang D. (2015). Progress Achieved on Natural ex situ Conservation of the Yangtze Finless Porpoise. IUCN SSC- Cetacean Specialist Group. Available online at: http://www.iucn-csg.org/index.php/2015/12/10/progress-achieved-on-natural-ex-situ-conservation-of-the-yangtze-finless-porpoise/ (Accessed April 21, 2018).

[B83] Wang K. Wang D. Zhang X. Pfluger A. Barrett L. (2006). Range-wide Yangtze freshwater dolphin expedition: The last chance to see Baiji? Conserv. Biol. 13, 418–424. 10.1065/espr2006.10.350 17120833

[B84] Whirledge S. Cidlowski J. A. (2010). Glucocorticoids, stress, and fertility. Minerva Endocrinol. 35, 109–125. 20595939 PMC3547681

[B85] Wright A. J. Soto N. A. Baldwin A. L. Bateson M. Beale C. M. Clark C. (2007). Do marine mammals experience stress related to anthropogenic noise? Int. J. Comp. Psychol. 20, 274–316.

[B86] Wu G. Leeuw J. D. Skidmore A. K. Prins H. H. T. Liu Y. (2007). Concurrent monitoring of vessels and water turbidity enhances the strength of evidence in remotely sensed dredging impact assessment. Water Res. 41, 3271–3280. 10.1016/j.watres.2007.05.018 17583768

[B87] Yang G. S. Ma C. D. Chang S. Y. (2009). Yangtze Conservation and Development Report 2009. Wuhan: Yangtze River Press.

[B88] Yang J. Xiao W. Kuang X. Wei Z. Liu R. (2000). Studies on the distribution, population size and the activity of *Lipotes vexillifer* and *Neophocaena phocaenoides* in Dongting Lake and Poyang Lake. Res. Environ. Yangtze Basin 9, 444–450.

[B89] Zhang K. (2007). Poyang Lake: Saving the Finless Porpoise. Available online at: http://www.chinadialogue.net/article/show/single/en/839-Poyang-Lake-saving-the- finless-porpoise (Accessed 6 April 2018).

[B90] Zhang X. Liu R. Zhao Q. Zhang G. Wei Z. Wang X. (1993). The population of finless porpoise in the middle and lower reaches of Yangtze River. Acta Theriol. Sin. 13, 260–270.

[B91] Zhao X. Barlow J. Taylor B. L. Pitman R. L. Wang K. Wei Z. (2008). Abundance and conservation status of the Yangtze finless porpoise in the Yangtze River, China. Biol. Conserv. 141, 3006–3018. 10.1016/j.biocon.2008.09.005

[B92] Zhong Y. Chen S. (2005). Impact of dredging on fish in Poyang Lake. Jiangxi Fish Sci. Technol. 1, 15–18.

